# Enthesitis in a 16-Year-Old Boy with M694V Mutation

**DOI:** 10.1155/2016/5869250

**Published:** 2016-12-04

**Authors:** Syert Luidolf Nienhuis, Robin Eric Westerbeek

**Affiliations:** Department of Radiology, Deventer Ziekenhuis, Deventer, Netherlands

## Abstract

*Introduction*. FMF (Familial Mediterranean Fever) is characterized by recurrent attacks of fever and articular pain. Enthesitis is the hallmark of pain in spondyloarthropathy. Literature suggests association of M694V mutation and enthesitis. We report a case of a 16-year-old boy with enthesitis and FMF.* Case Presentation*. A 16-year-old boy of Turkish origin with a history of FMF presented with localized tenderness of the heel and severe disability. MRI showed an enthesitis of the plantar fascia. Standard treatment of FMF and enthesitis was not successful. After referral to a university hospital and expert opinion of a professor in rheumatology, this enthesitis should be treated as an enthesitis related arthritis. With this treatment, our patient fully recovered 8 months after the onset of the disease symptoms.* Conclusion*. M694V mutation related enthesitis should be considered in FMF patients with enthesitis. We would suggest treatment for enthesitis related arthritis in similar cases. This is of clinical importance because the treatment is different from treatment of enthesitis or articular pain caused by FMF.

## 1. Introduction

Enthesopathy is defined as inflammation of the insertions of tendons, ligaments, or joint capsules. Although entheses are located in different body parts, the lower extremities are the most commonly affected regions. Clinical presentation differs from asymptomatic findings on imaging to severe disability [1, 2].

Familial Mediterranean Fever (FMF), also known as paroxysmal polyserositis, is an autosomal recessive disease affecting mainly Middle Eastern and eastern Mediterranean populations. It is characterized by recurrent fever and serosal inflammation, causing thoracic, abdominal, and articular pain. The* MEFV gene* is responsible for this disease. More than 300 mutations have been identified. The* MEFV gene* codes for a protein called pyrin [3, 4].

We report a case of a 16-year-old boy with enthesitis and Familial Mediterranean Fever.

## 2. Case Report

A 16 year-old-boy of Turkish origin presented with severe pain at the heel. In the past, he was diagnosed with Familial Mediterranean Fever. Family history included a sister with FMF. At the outpatient paediatric clinic, the patient had pain for about 6 weeks. At physical examination, there was tenderness at the calcaneal bone and an antalgic gait. Laboratory tested HLA B27 negative. Nonsteroidal anti-inflammatory drug (NSAID) and inlays showed no improvement of disability. Standard medication for musculoskeletal pain caused by FMF such as colchicine did not improve his symptoms and disability.

### 2.1. Imaging

**Figure 1:**
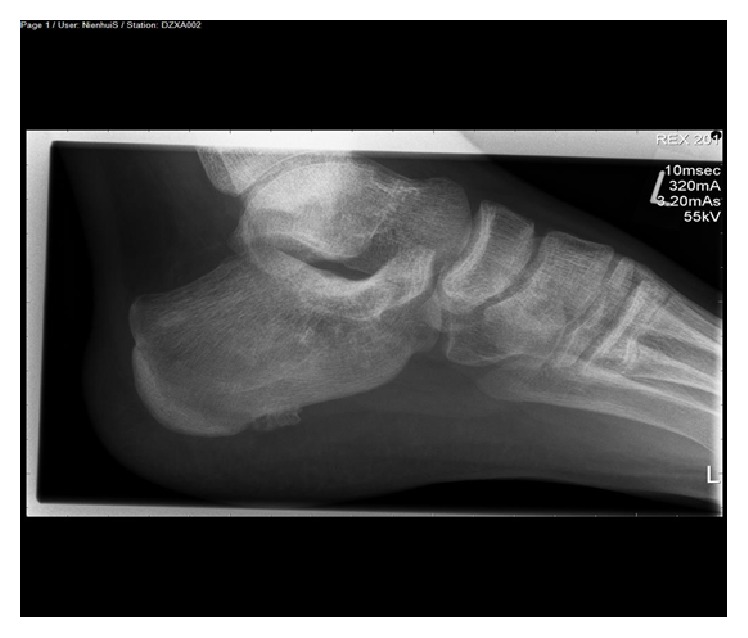
Plain radiograph: lateral view of the left foot: osteochondroma located at the plantar side of the calcaneal bone.

**Figure 2:**
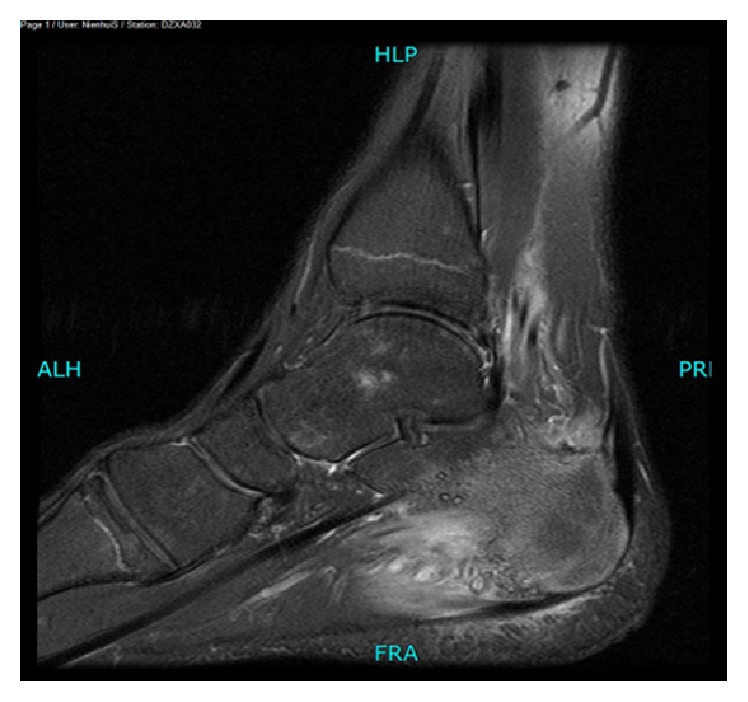
MRI T2 fat suppression: sagittal view of the left foot: high signal intensity of the soft tissue close to the plantar fascia. Secondary edema of bone marrow in the calcaneal bone.

**Figure 3:**
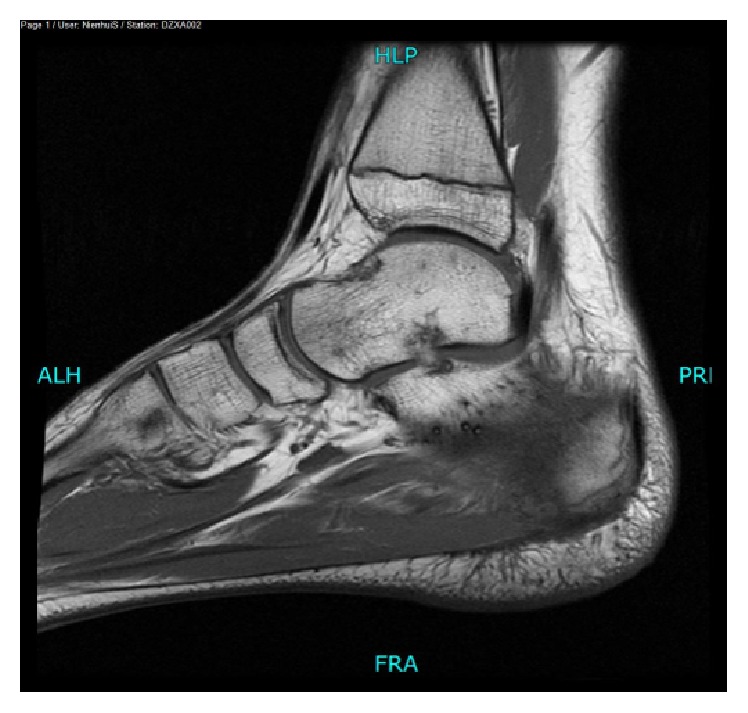
MRI T1: sagittal view of the left foot: enthesophyte at the insertion of the plantar fascia.

**Figure 4:**
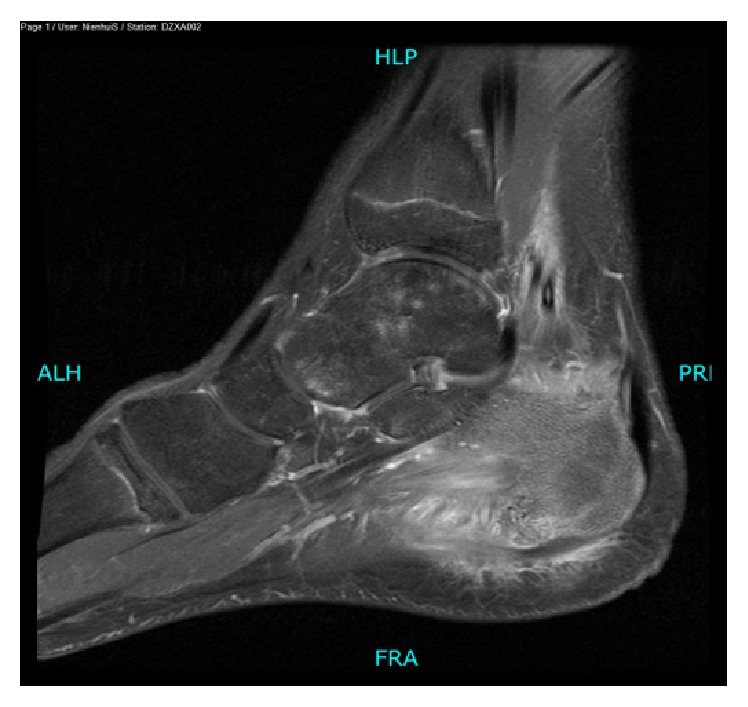
MRI T1 fat suppression after gadolinium: sagittal view of the left foot: attenuation of soft tissue near the enthesophyte.

Plain radiograph showed an osteochondroma located at the plantar side of the calcaneal bone (Figure 1). An additional MRI was performed because of the severe disability. T2 fat suppression images showed high signal intensity of the soft tissue close to the insertion of the plantar fascia (Figure 2). T1 weighted images showed an enthesophyte (Figure 3). No relationship was seen between high signal intensity of the soft tissue and the osteochondroma. T1 fat suppression images with gadolinium showed attenuation of soft tissue near the enthesophyte (Figure 4). There was secondary calcaneal bone marrow edema, related to the soft tissue abnormalities. In conclusion, MRI showed an enthesitis of the plantar fascia.

### 2.2. Treatment

An orthopaedic surgeon was consulted for the treatment of plantar fasciitis. Immobilisation, NSAID, and inlays did not have any effect. Therefore, a cast immobilisation for 6 weeks was prescribed. A second opinion in university hospital did not alter the diagnosis of plantar fasciitis.

### 2.3. After 3 Months

No clinical improvement was seen after 3 months of therapy. MRI examination was repeated and there were no alterations in the diagnosis of plantar fasciitis.

### 2.4. Literature

We did a literature study and this resulted in an article of Tufan et al. [5], in which an association was reported between a M694V mutation in* MEFV gene* and enthesopathy. Therefore, genetic analysis was done and revealed a M694V mutation in our case. After a referral to a university hospital with more experience with FMF patients and expert opinion of a professor in rheumatology, the enthesitis in this case should be treated like an enthesitis related arthritis (ERA).

The treatment was changed to a combination of NSAID and sulfasalazine (disease modulating antirheumatic drug, DMARD), our patient showed clinical improvement. The enthesitis had been resolved after 8 months of disability. After one year, our patient is still without any physical symptoms.

## 3. Discussion

Inflammation at entheses is the clinical hallmark in spondyloarthropathies. Ineffective treatment of enthesitis will result in disease progression. Therefore, early diagnosis is mandatory [6]. In case of plantar fascia enthesitis, standard treatment is rest and activity modulation, NSAIDs, inlays, physical therapy, and plantar fasciotomy [7].

FMF is an autoinflammatory disease and is characterized by recurrent attacks of fever and polyserositis leading to abdominal, thoracic, or articular pain. The* MEFV gene* is responsible for the disease. The most effective treatment for FMF patients is colchicine to decrease frequency and severity of crises [3, 4].

In our patient, there was an enthesitis and previous history of FMF. Tufan et al. investigated the association between FMF and enthesopathy. Tufan et al. showed no significant relation between FMF and enthesitis. However, analysis of several mutations in the* MEFV gene* showed a significant association of M694V mutation with enthesitis. Other mutations do not have any association [5]. Enthesitis is strongly associated with HLA B27 but is also seen in HLA B27 negative patients [8]. Gülhan et al. suggests that MEFV mutations may represent a susceptibility factor for ERA in the populations of the eastern Mediterranean [9].

In our case, complaints were misdiagnosed as symptoms caused by FMF and as standard plantar fasciitis without improvement of standard treatment. The intimate relationship between enthesis, organs, and synovial cavities and the presence of enthesis organ components in joint capsules may have important implications for understanding the clinical pattern [10, 11]. In our case genetic analysis showed a M694V mutation and a negative laboratory test for HLA B27. Our patient might have developed an independent ERA or an M694V mutation related enthesitis in combination with a history of FMF. According to the international league of associations of rheumatology, this enthesitis is a subtype of juvenile idiopathic arthritis and could be considered as an enthesitis related arthritis [8]. Treatment of ERA is different in comparison to standard enthesitis or musculoskeletal pain caused by FMF, so this is clinically very important. Treatment regimens of ERA are NSAIDs, DMARDs, or biological agents [12].

In literature, only one case is reported of enthesopathy and arthropathy syndrome with FMF in a 20-year-old patient with a history of inflammatory back pain [13]. Future studies should further investigate the association between enthesitis and the M694V mutation and the treatment of this pathology. It is important to consider M694V mutation related enthesitis in whom enthesitis is seen on imaging and has a history of FMF, even in areas where FMF is not that common.

In conclusion, M694V mutation related enthesitis should be considered in FMF patients with an enthesitis. We would suggest treatment for enthesitis related arthritis in similar cases. This is of clinical importance, because different treatment is needed in comparison to standard enthesitis or musculoskeletal articular pain in FMF.
